# *Rhodococcus equi* Infection after Alemtuzumab Therapy for T-cell Prolymphocytic Leukemia

**DOI:** 10.3201/eid1312.061559

**Published:** 2007-12

**Authors:** Jan J. Meeuse, Herman G. Sprenger, Sander van Assen, Dominique Leduc, Simon M.G.J. Daenen, Jan P. Arends, Tjip S. van der Werf

**Affiliations:** * University Medical Center Groningen, Groningen, the Netherlands; † Centre Hospitalier Intercommunal Annemasse-Bonneville, Bonneville, France

**Keywords:** Rhodococcus equi, Alemtuzumab, T-PLL, dispatch

## Abstract

*Rhodococcus equi*, mainly known from veterinary medicine as a pathogen in domestic animals, can also cause infections in immunocompromised humans, especially in those with defects in cellular immunity. Alemtuzumab, an anti-CD52 monoclonal antibody, causes lymphocytopenia by eliminating CD52-positive cells. We report a patient in whom *Rhodococcus equi* infection developed after alemtuzumab therapy.

*Rhodococcus equi* is a soil-borne, asporogenous, nonmotile, obligate aerobe; it is also a facultative, intracellular, gram-positive microorganism that can survive inside macrophages, the characteristic considered the basis for its pathogenicity (*1*). In foals and other domestic animals, it is an important respiratory and intestinal pathogen (*2*). Human infection with *R. equi* is rare but can occur in immunocompromised patients, especially those who have HIV infection and a CD4^+^ cell count <100 × 10^6^/L (*3*). The clinical manifestations are diverse, although 80% of patients have some pulmonary involvement (*3*). In recent decades, an increased incidence of *R. equi* infections in humans has been reported. This increase may be due to the rising number of immunocompromised patients as a result of increasing numbers of organ transplantations and intensified antitumor chemotherapy. We describe a patient with T-prolymphocytic leukemia (T-PLL) in whom a febrile disease with lung abscess due to *R. equi* developed 10 weeks after the complete remission of leukemia was induced by chemotherapy combined with alemtuzumab.

## Case Report

A 68-year-old man with T-PLL (leukocyte count 174.5 × 10^9^/L, 96% lymphoid cells) was treated with chemotherapy consisting of cyclophosphamide, doxorubicin, vincristine, and prednisolone every 2 weeks (CHOP14), in combination with alemtuzumab 30 mg subcutaneously on days 1, 5, and 9 of each cycle. This combined therapy was well tolerated. Complete cytologic and immunohistochemical remission was confirmed by blood and bone marrow examination 2 weeks after the latest chemotherapy treatment. Ten weeks later, the patient experienced flu-like symptoms and had a fever of 38.9°C. One week earlier, the antimicrobial prophylaxis, which consisted of valacyclovir, 500 mg 2 times/day, and trimethoprim-sulfamethoxazole, 960 mg 3 times/week, had been stopped, although the alemtuzumab-induced lymphocytopenia was still present (leukocytes 7.2 × 10^9^/L, 84% neutrophils, 0.6% lymphocytes). Outpatient evaluation showed 2 lung abscesses. From 3 consecutive blood cultures and from the bronchoalveolar lavage fluid, a gram-positive bacillus with mucoid growth was isolated and identified as *R. equi* (API Coryne, bioMérieux, Marcy l’Etoile, France). The isolated strain was resistant to β-lactam antimicrobial drugs and trimethoprim-sulfamethoxazole and susceptible to aminoglycosides, tetracyclines, fluoroquinolones, glycopeptides, erythromycin, and rifampin. Treatment with moxifloxacin and rifampin was begun. After 3 weeks of treatment, fever developed in the patient again. Blood cultures grew *R. equi*. The patient was admitted to the hospital for intravenous treatment with imipenem/cilastatin, 500 mg/500 mg 3 times/day, and vancomycin, 1.5 g once a day. A computed tomographic scan of the chest showed progression of the pulmonary abscesses and mediastinal lymphadenopathy. Clarithromycin, 500 mg 2 times/day, was added, and the vancomycin was increased to 2 g once a day, which resulted in clinical improvement. Purple, subcutaneous, oval lesions, 2–3 cm in diameter and not painful to palpation, were seen on the upper portion of both legs. Pathologic examination of these lesions after biopsy showed suspected localization of T-PLL. *R. equi* could not be demonstrated in these skin lesions by either pathologic or microbiologic examination. After 2 weeks of receiving intravenous antimicrobial drugs, the patient was discharged with oral rifampicin, 600 mg once a day; ciprofloxacin, 750 mg twice/day; and azithromycin, 500 mg once a day.

He was readmitted to our hospital 9 weeks later because he had become dyspneic and febrile. Evaluation showed pleural effusion on the right side. Progression of the T-PLL was also diagnosed. After 1 week’s incubation of the pleural fluid, mucoid nonpigmented colonies were growing, consisting of gram-positive coccoid rods, which were catalase positive. *Rhodococcus* infection was suspected and confirmed by 16S rDNA sequencing without further conventional identification. The isolate showed intermediate susceptibility to ciprofloxacin (MIC 0.75 mg/L), moxifloxacin (MIC 0.5 mg/L), and erythromycin (MIC 1.5 mg/L). Drainage of the pleural fluid resulted in a trapped lung due to pleural thickening. A pleurectomy was considered but was refused by the patient, considering his poor overall prognosis based on the relapse of T-PLL. On his request, the antimicrobial drugs were stopped, and he went home with palliative treatment consisting of morphine and prednisone. He died 3 months later. Overall, he had been treated with antimicrobial agents for 19 weeks.

## Conclusions

The described patient acquired a *R. equi* infection during alemtuzumab-induced lymphocytopenia. *R. equi* infection is predominantly airborne, acquired through the respiratory tract. Exposure to domestic animals, such as horses and pigs, may play a role in acquisition of this organism. The patient denied any such contact, as do two thirds of all patients infected with *R. equi* (*3*).

Alemtuzumab is approved as a second-line treatment in chronic lymphatic leukemia and is increasingly used in therapeutic trials for T-cell malignancies. It is a recombinant DNA-derived, humanized monoclonal antibody directed against CD52 (*4*). CD52 is a membrane glycoprotein expressed mainly by lymphocytes, especially T cells. Alemtuzumab causes lysis of these cells by binding to CD52, resulting in lymphocytopenia, which can persist for up to 320 days after treatment (*5*). While the patient is experiencing lymphocytopenia, prophylaxis with an antiviral agent and trimethoprim-sulfamethoxazole are mandatory to prevent the most frequent opportunistic infections (*6*). Reduced cellular immunity is known to predispose to infection with *R. equi* (*7*). Primary prophylaxis is not routinely recommended because no data are available to support its efficacy and because the infection is rare (*3*). Due to variable susceptibility to trimethoprim-sulfamethoxazole, the prophylaxis regimen used after alemtuzumab therapy will not prevent *R. equi* infection in all patients, as this case illustrates.

Standard treatment regimens for *R. equi* infections have not been established. Weinstock and Brown advised intravenous therapy with 2 or 3 drug regimens that include vancomycin, imipenem, aminoglycosides, ciprofloxacin, rifampin, or erythromycin (*3*). This recommendation was based on in vitro susceptibility data and published case reports. Treatment should preferably be guided by susceptibility testing. After clinical improvement (usually after 2–4 weeks), oral antimicrobial agents can then be substituted and continued until all culture results are negative and the patient’s symptoms and signs have resolved. A minimum of 6 months of antimicrobial drug therapy is typically required for immunocompromised patients with pulmonary, bone and joint, or central nervous system infections (*3*).

Our patient started treatment with oral antibiotics, guided by susceptibility tests. Although moxifloxacin and rifampin are known for their good oral resorption, and despite initial clinical improvement, progression of the infection was apparent by the clinical course. Susceptibility testing was not performed at this time, but testing later in the clinical course suggested a decrease in susceptibility by the *R. equi* strain to the antimicrobial agents given.

After this regimen failed, intravenous therapy with 3 antimicrobial drugs was instituted. However, also this strategy ultimately failed. Apart from persistence of bacilli due to poor penetration at the site of infection, and the possible development of resistance, this lack of response is likely due to persistent lymphocytopenia resulting from previous treatment with alemtuzumab and progression of T-PLL.

In summary, longstanding alemtuzumab-induced lymphocytopenia is the most likely cause of the uncontrollable opportunistic *R. equi* infection in the described patient. This case illustrates the therapeutic challenges of this kind of infection in severely immunocompromised patients.
